# Dual Transcriptomic Analysis Reveals a Delayed Antiviral Response of *Haliotis diversicolor supertexta* against Haliotid Herpesvirus-1

**DOI:** 10.3390/v11040383

**Published:** 2019-04-24

**Authors:** Chang-Ming Bai, Shu-Min Zhang, Ya-Na Li, Lu-Sheng Xin, Umberto Rosani, Chong-Ming Wang

**Affiliations:** 1 Key Laboratory of Maricultural Organism Disease Control, Ministry of Agriculture, Qingdao Key Laboratory of Mariculture Epidemiology and Biosecurity, Yellow Sea Fisheries Research Institute, Chinese Academy of Fishery Sciences, Qingdao 266071, China; baicm@ysfri.ac.cn (C.-M.B.); zhangshumin1223@foxmail.com (S.-M.Z.); lyn19933@163.com (Y.-N.L.); xinls@ysfri.ac.cn (L.-S.X.); 2 Laboratory for Marine Fisheries Science and Food Production Processes, Qingdao National Laboratory for Marine Science and Technology, Qingdao 266237, China; 3 Department of Fisheries and Life Science, Dalian Ocean University, Dalian 116023, China; 4 Department of Fisheries, Tianjin Agriculture University, Tianjin 300380, China; 5 Department of Biology, University of Padua, 35121 Padua, Italy; 6 Alfred Wegener Institute (AWI)—Helmholtz Centre for Polar and Marine Research, Wadden Sea Station Sylt, 25992 List, Germany

**Keywords:** abalone, transcriptome, immune-related genes, apoptosis, HaHV-1

## Abstract

Haliotid herpesvirus-1 (HaHV-1) is the first identified gastropod herpesvirus, causing a highly lethal neurologic disease of abalone species. The genome of HaHV-1 has been sequenced, but the functions of the putative genes and their roles during infection are still poorly understood. In the present study, transcriptomic profiles of *Haliotis diversicolor supertexta* at 0, 24 and 60 h post injection (hpi) with HaHV-1 were characterized through high-throughput RNA sequencing. A total of 448 M raw reads were obtained and assembled into 2.08 × 10^5^ unigenes with a mean length of 1486 bp and an N50 of 2455 bp. Although we detected increased HaHV-1 DNA loads and active viral expression at 24 hpi, this evidence was not linked to significant changes of host transcriptomic profiles between 0 and 24 hpi, whereas a rich immune-related gene set was over-expressed at 60 hpi. These results indicate that, at least at the beginning of HaHV-1 infection, the virus can replicate with no activation of the host immune response. We propose that HaHV-1 may evolve more effective strategies to modulate the host immune response and hide during replication, so that it could evade the immune surveillance at the early stage of infection.

## 1. Introduction

Haliotid herpesvirus-1 (HaHV-1), one of the most devastating viral pathogens of wild and cultivated abalones, is the first identified herpesvirus that infects gastropods [1,2]. HaHV-1 infection was first reported in 2003 in Taiwan, where mass mortalities of cultivated *Haliotis diversicolor supertexta* were reported [1]. Subsequently, a similar disease was described in the blacklip abalone (*Haliotis rubra*) and in the greenlip abalone (*Haliotis laevigata*) in Australia in 2005 [3]. Two recent studies back-dated the appearance of HaHV-1 infection to 1999 in *H. diversicolor supertexta* populations cultivated in Southern China [4,5]. Histological lesions of HaHV-1 infection were characterized by necrotizing ganglioneuritis and, therefore, this disease was named Abalone Viral Ganglioneuritis (AVG). Although the complete genome of HaHV-1 has been sequenced, most of its putative proteins still have unknown functions [2]. Phylogenetic analysis based on two conserved proteins, the DNA packaging ATPase subunit of a (putative) terminase and the DNA polymerase, showed that HaHV-1 is closely related to Ostreid herpesvirus-1 (OsHV-1), the first herpesvirus discovered to infect mollusks [6,7], whereas HaHV-1 is only distantly related to the other herpesviruses [2]. This great divergence has prevented the possibility to infer gene functions by homology-based prediction methods [7], greatly limiting the knowledge on these two herpesviruses.

Herpesviruses are large dsDNA viruses, often evolved with their host over a long period of time resulting in a strict host–virus co-adaptation [8]. Vertebrate herpesviruses used many strategies to circumvent the host immune responses or delay them [9,10,11,12]. The infection mechanisms and the viral and host key genes involved during the infection process have been characterized for a number of vertebrate herpesviruses, and they were used to develop preventative or therapeutic measures [9,13]. Conversely, the antiviral responses of mollusks need further work to be extensively understood [14,15,16,17,18]. The lack of continuous molluscan cell lines supporting viral growth and replication in vitro greatly hampered the application of the classical virology approaches to these viruses [14]. Molecular approaches and the introduction of next-generation sequencing (NGS) techniques have increased the possibility to understand viral genomic diversity and transcriptional landscape of OsHV-1 [19,20,21,22,23]. Dual transcriptomic analysis of the cross-talk between OsHV-1 and bivalve mollusks active infection processes have been also carried out [16,24,25,26,27]. These studies revealed a rich set of genes and pathways in bivalves, especially in *Crassostrea gigas*, involved in the immune defense against OsHV-1 infection [14,16,28,29,30]. One of the highly noticeable pathways revealed by these studies is apoptosis [16,18,25], which also plays an important role in eliminating herpesvirus-infected cells in vertebrates [31,32,33]. To evade elimination by apoptosis and guarantee viral propagation, herpesviruses have evolved various strategies to suppress host cell apoptosis. OsHV-1 genome encodes several putative inhibitors of apoptosis (IAPs) capable of suppressing host apoptosis and allowing its own replication [7,28]. Moreover, some key anti-apoptotic factors regulating apoptotic signaling pathway were also over-expressed during OsHV-1 infection [16,25,26,28]. The apoptosis signaling pathway is certainly not isolated, many other key components associated with antiviral responses, including interferon pathways, toll-like pathways, and oxidative bursts have been reported in *C. gigas* [14,16,25]. Another remarkable observation during the interaction between *C. gigas* and OsHV-1 was that the early antiviral response (before 24 h after infection) was important to successfully counteract the viral infection [27].

Although it is expected that several other herpesviruses that infect invertebrates exist [2], HaHV-1 and OsHV-1 represent the most destructive viral disease of mollusks worldwide [34]. HaHV-1 was less studied compared to OsHV-1, and no data are available to characterize the molecular mechanisms underlying the viral pathogenesis and abalone’s countermeasures. In this study, we characterized the dual transcriptome changes of HaHV-1 and *H. diversicolor supertexta* at 0, 24, and 60 h post injection (hpi). We provided an annotated transcriptome of *H. diversicolor supertexta* and the first insight into its antiviral pathways. Our analysis indicated an unexpected delay in the immune response to HaHV-1 infection. Globally, these transcriptomic data provided a valuable resource for the gastropod research community.

## 2. Materials and Methods

In April 2016, approx. 400 *H. diversicolor supertexta* specimens (size range from 49.73 to 58.24 mm, *n* = 30) were transferred by air from Xiamen to our aquaculture facilities in Qingdao (YSFRI). The abalones were derived from hatchery produced seeds reproduced from 50 pairs of parental abalones. The abalones were cultivated in 50-L tanks (approximately 40 abalones per tank) supplied with aerated, sand-filtered seawater, and fed with seaweed (*Laminaria japonica*). The water temperature was maintained at approximately 18 °C, and half-changed daily during the acclimation period of two weeks. At the end of acclimation, 30 *H. diversicolor supertexta* were selected randomly and tested to be negative for HaHV-1 DNA.

For viral inoculum preparation, the standard protocol used for OsHV-1 and described in [35] was employed except for the use of natural seawater in all dilution steps. A single infected *H. diversicolor supertexta* (HaHV-1-CN2003) was used as a source of HaHV-1. This specimen was originally collected during abnormal mortality outbreaks in Guangdong Province, China in 2003 [5]. Tissue homogenates for negative controls were prepared using HaHV-1 PCR negative *H. diversicolor supertexta* animals applying the same protocol. A small aliquot of each homogenate (200 µL) was used for HaHV-1 DNA detection and quantification, as described above.

For experimental infection, abalones were “anesthetized” (myo-relaxation) with 10 g/L of MgCl2 and randomly divided into challenged and negative control groups composed by 180 and 70 individuals, respectively. Once the abalones were relaxed, 100 μL of viral inoculums (1.0 × 10^4^ copies of HaHV-1 DNA/μL) or control tissue homogenates were injected into the pedal muscle using 1 mL micro-syringes equipped with 18-g needles. A total of 150 challenged abalones distributed in 50 L tanks (50 abalones per tank) and 40 control abalones maintained in one 50 L tank were used for the time course experiment. The remaining 30 individuals per group were maintained in 18 L tanks (10 abalones per tank) and used for monitoring of mortality rates. Mortality was monitored every 12 h after infection, and dead abalones were removed from the tank.

At each time point (0, 12, 24, 30, 36, 48, 60, and 72 h post injection, hpi), 6 challenged (2 abalones per tank) and 3 control abalones were randomly sampled. The mantle of each abalone was immediately dissected and divided into 2 pieces for DNA and RNA extraction. Dead abalones were collected from each tank and a piece of the mantle was sampled for DNA extraction and HaHV-1 DNA quantification. The DNA extraction was performed using the TIANamp^TM^ Marine Animals DNA Kit (Tiangen Biotech, Beijing, China), according to the manufacturer’s protocol. The purity and quantity of the isolated DNA were determined with a Nanodrop 2000 spectrophotometer (Thermo Fischer Scientific, Waltham, USA). HaHV-1 DNA quantification was carried out by quantitative PCR (qPCR) targeting ORF66 (DNA polymerase) adapted from the World Organization for Animal Health (OIE) Manual of Diagnostic Tests for Aquatic Animals, 2017, and fully described by Bai et al (2019). We estimated the HaHV-1 infection burden of each sample as the mean genomic equivalent (GE) score (ng^−1^ of total DNA).

Mantle tissues of 3 abalones collected at 0, 24, and 60 hpi were selected for RNA-seq according to the measured trend of viral infection burden. The 9 RNA samples were designated as MA00h (1–3), MA24h (1–3), and MA60h (1–3) and sent to Beijing Novogene Technology Co. Ltd. (Beijing, China). for RNA extraction and high-throughput sequencing using the Illumina Hiseq platform (Illumina Inc. San Diego, USA). A total amount of 1.5 µg RNA per sample was used as the input material for the library preparations, which were generated using the NEBNext^®^ Ultra™ RNA Library Prep Kit for Illumina^®^ (NEB, Ipswich, USA) following the manufacturer’s recommendations. Index codes were added to attribute the reads to each sample. Briefly, mRNA was purified from total RNA using poly-T oligo-attached magnetic beads (NEB). Fragmentation was carried out using divalent cations under elevated temperature in a NEB NextFirst^®^ Strand Synthesis Reaction Buffer (5×). First strand cDNA was synthesized using random hexamer primers and M-MuLV Reverse Transcriptase (RNase H, NEB). Second strand cDNA synthesis was subsequently performed using DNA Polymerase I and RNase H (NEB). The remaining overhangs were converted into blunt ends via exonuclease/polymerase activities (NEB). After adenylation of the 3’ ends of the DNA fragments, NEBNext^®^ adaptors with hairpin loop structure were ligated to DNA. To select fragments of preferentially 250~300 bp in length, the library fragments were purified with AMPure XP system (Beckman Coulter, Brea, USA). Then 3 µL USER Enzyme (NEB) was used with size-selected, adaptor-ligated cDNA at 37 °C for 15 min followed by 5 min at 95 °C before PCR. Subsequently, PCR was performed with Phusion High-Fidelity DNA polymerase (NEB), Universal PCR primers, and Index (X) Primer (NEB). At last, PCR products were purified with AMPure XP system (Beckman Coulter) and library quality was assessed on the Agilent Bioanalyzer 2100 system (Agilent Technologies, Palo Alto, CA, USA). The clustering of the index-coded samples was performed on a cBot Cluster Generation System using TruSeq PE Cluster Kit v3-cBot-HS (Illumina Inc.) according to the manufacturer’s instructions and sequencing was carried out on an Illumina Hiseq platform (2 × 150 paired-end reads).

Raw Illumina reads were trimmed to remove adaptor sequences, low-quality positions (Phred quality of 20 was used as a cut-off), and reads containing poly-n and reads shorter than 50 bp. The resulting high-quality (HQ) reads were deposited at the NCBI SRA Database and used in the downstream analyses. Since there is no reference genome available for *H. diversicolor supertexta*, and only 30–32 % of the reads can be mapped to the only available genome of Haliotis family [36], we applied a reference-independent strategy. *De-novo* transcriptome assembly was performed with the Trinity assembler (v.2.4.0) [37] setting min_kmer_cov to 2 and all other parameters to defaults. The obtained unigenes were functionally annotation searching for similar hits in seven databases: Nr (NCBI non-redundant protein sequences, *e*-value = 1e^−5^), Nt (NCBI non-redundant nucleotide sequences, *e*-value = 1e^−5^), KO (KEGG Orthology database, *e*-value = 1e^−10^), GO (Gene Ontology, *e*-value = 1e^−6^), KOG (euKaryotic Ortholog Groups, *e*-value = 1e^−3^), Pfam (Protein family, *e*-value = 1e^−2^), and Swiss-Prot (*e*-value = 1e^−5^).

To produce an expression profile of the HaHV-1 genes, the clean reads were mapped on the HaHV-1 genome (GenBank No. KU096999) with the recently improved annotation [38], applying 0.8 for both length and similarity mapping parameters (CLC Genomics, v.11, Qiagen, Hilden, Germany). The total number of mapped reads was used as a proxy of the HaHV-1 expression value for each of the annotated genes and compared between the 3 experimental groups. In order to classify the viral genes as early or late-expressing genes, we considered the expression ratios between the time points. Briefly, we measured the expression ratio between 24 hpi and 0 hpi, and between 60 hpi and 24 hpi for each viral gene, and compared these two values in order to classify the viral genes as ‘early-expressed’ (the ones with a 24/0 value higher than 10 timed the 60/24 value), ‘late-expressed’ (the ones with a 60/24 value higher than the 24/0 value), or stable (the remaining expressed genes, i.e., the ones with a 24/0 value slightly higher than the 60/24 value).

Differential expression analysis was performed using the DESeq R package (v.1.10.1). DESeq provides statistical routines for determining differential expression in digital gene expression data using a model based on the negative binomial distribution. The resulting *p* values were adjusted using the Benjamini and Hochberg’s approach for controlling the false discovery rate. Genes with an adjusted *p*-value < 0.05 found by DESeq were considered as differentially expressed and a pathway enrichment analysis based on the KEGG database annotations was performed on them. GO enrichment analysis was carried out with GOseq R packages based Wallenius non-central hyper-geometric distribution, which can adjust for gene length bias in differentially expressed genes (DEGs) [39]. Finally, KOBAS software was used to test the statistical enrichment of differential expression genes in KEGG pathways [40].

To validate the expression values obtained by RNA-seq analysis, 8 DEGs were selected for reverse transcription quantitative PCR (RT-qPCR) conformation. These DEGs meet the following 3 criteria: (i) involved in immune-related pathways, (ii) have high/middle expression levels, (iii) perfect PCR efficiencies ranged from 95 % to 100 %. Primers were designed with Primer Premier 5 (Table S1) [41]. First strand cDNA was synthesized from 2 μg of total RNA with reverse transcriptase (Takara, Shiga, Japan) and random primers, and the resulting products were used as a template for qPCR analysis. qPCR was performed on CFX Connect™ Real-Time System (Bio-Rad Laboratories, Inc. Hercules, USA) with FastStart Essential DNA Green Master (Roche Diagnostics, Risch-Rotkreuz, Swiss). qPCR reactions were performed in 20 μL reaction system under the following thermal cycling conditions: 1 cycle of 95 °C for 10 min, followed by 40 cycles of 95 °C for 10 s, and 61 °C for 33 s and a melt curve step (from 65 °C, gradually increasing 0.5 °C/s to 95 °C, with acquisition data every 1 s). Cytc1 were used as the internal standards to normalize the relative expression levels among samples (Zhang SM et al. [42]). All reactions were performed in triplicates and the expression values were calculated as the mean of relative mRNA expression using the 2^−ΔΔCT^ method [43].

## 3. Results and Discussion

### 3.1. Abalone Mortalities and Viral DNA Quantification

We tested the susceptibility of *H. diversicolor supertexta* to HaHV-1 by performing a controlled infection trial. While no mortality occurred during the acclimation period, after HaHV-1 infection the first mortality occurred at 48 hpi (6.7% mortality) and developed quickly for the challenged group, reaching 100 % of mortality at 96 hpi. The accumulative mortality rates were 13.3, 76.7, and 80.0 % at 60, 72, and 84 hpi, respectively. The average amount of viral DNA was estimated as 1.05 × 10^6^ copies ng^−1^ of total DNA in the mantles of dead abalone. In the HaHV-1 challenged group, the viral DNA exponentially increase after injection and reached averages of 45.9, 1.5 × 10^6^, 4.3 × 10^6^, 5.6 × 10^6^, 2.2 × 10^7^, 6.5 × 10^7^, and 8.0 × 10^6^ at 12, 24, 30, 36, 48, 60, and 72 hpi, respectively. No mortality or viral DNA was detected in the negative controls at the completion of the experiment. The rapid increase of viral DNA, as well as the high mortality rates, indicated that the experimental infection was successful and that the abalone family we used was highly susceptible to HaHV-1 infection.

### 3.2. Transcriptome Assembly and Functional Annotation

**Figure 1:**
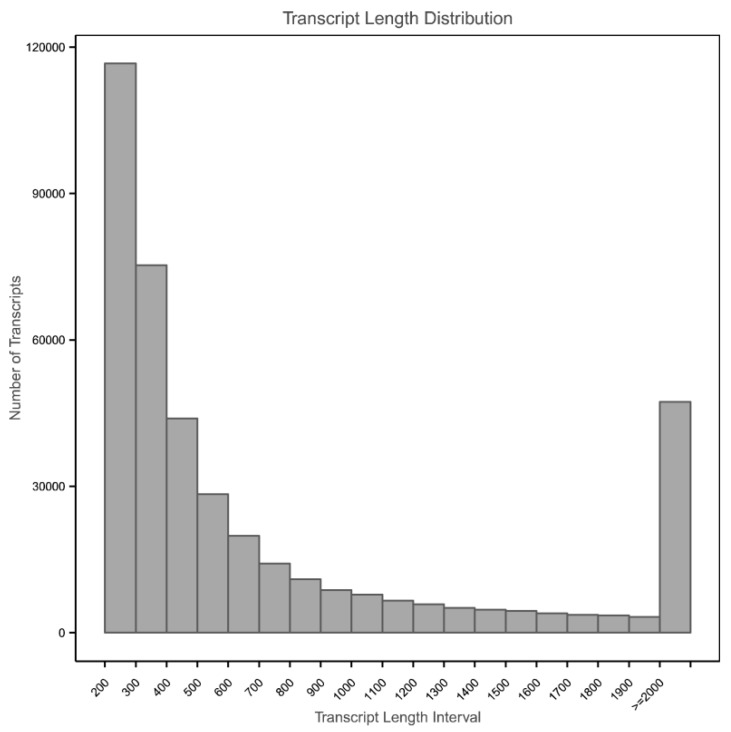
Length distribution of assembled unigenes.

A total of 448 M raw reads were generated from the nine sequenced libraries, with 155 M, 143 M, and 150 M reads from MA00h, MA24h, and MA60h groups, respectively (Table 1, NCBI SRA accession number: PRJNA514990 and PRJNA471241). After removing low-quality reads, we de novo assembled 435 M of clean reads. into 2.08 × 10^5^ unigenes, showing a mean length of 1486 bp and an N50 of 2455 bp. Among these, 5.25 × 10^4^ unigenes (25.2%) were in the length range of 200 to 500 bp, 6.02 × 10^4^ unigenes (28.9%) were between 500 to 1000 bp, 4.82 × 10^4^ unigenes (23.2%) were between 1 to 2 kb, and 4.72 × 10^4^ unigenes (22.7%) were longer than 2 kb (Figure 1).

Since there is no reference genome available for *H. diversicolor supertexta*, or for relative species, the assembled unigenes were subjected to functional annotation by matching them against Nr, NT, KO, Swiss-prot, Pfam, GO, and KOG databases. As a result, 131,904 unigenes (63.37%) were annotated in at least one database, while 6029 unigenes (2.89%) were annotated in all the seven databases. The matching rates for the specific database were listed in Table 2. Regarding the homology search against the Nr database, 26.1% of unigenes had top matches with the owl limpet (*Lottia gigantea*), followed by *C. gigas* (21.9%), California sea hare (*Aplysia californica*) (17.3%), purple sea urchin (*Strongylocentrotus purpuratus*) (1.9%), Florida lancelet (*Branchiostoma floridae*) (1.7%), or with other species (31.0%) (Table S2). These annotation results mirrored the few sequence information available for the Haliotidae family, since the only transcriptome dataset of *H. diversicolor supertexta* was produced to investigate early development stages (larva and spat) [44]. Therefore, the transcriptomic data generated in the present study will enrich the available resources of this species and will contribute to disclose the abalones’ immune-related pathways.

In order to investigate the immune system of *H. diversicolor supertexta*, we further identified genes and pathways involved in immunity. GO classification analysis revealed that 1870 and 22,556 unigenes belong to “immune system process” and “response to stimulus”, respectively (Table S3), while KEGG pathways analysis revealed 16 immune-related pathways including 1694 unigenes (Table S4). Additionally, several pathways classified under the general term of as “signal transduction” are also known to be involved in immune processes of mollusks, like the “HIF-1 signaling pathway”, “Jak-STAT signaling pathway”, “NF-kappa B signaling pathway”, and “PI3K-Akt signaling pathway”.

### 3.3. Analysis of the HaHV-1 Expression Profile

Read mapping on the HaHV-1 genome identified 1431, 141,047, and 5,872,081 viral reads in the 0, 24 and 60 hpi samples, respectively. The presence of read mapping on the HaHV-1 genome even in the 0 hpi samples (before injection) is due to their mapping to low-complexity repeats on the viral genome. Total mapped reads per gene, used as a proxy of viral gene expression levels, exhibited negligible values at 0 hpi (0–39), increased values at 24 hpi (1–9012, median 205), and reached the highest values for most of the annotated genes at 60 hpi (1–175,765, median 9372, Table S5). Although the viral expression appeared to diffuse along the whole HaHV-1 genome both at 24 and 60 hpi, we attempted to classify early and late viral genes. To do this, we measured the expression ratios for each gene between the time points. This analysis revealed only 8 HaHV-1 genes with a prevalent ‘early’ expression trend, namely 7 ORFs with unknown function and one IAP. Most of the HaHV-1 ORFs exhibited somewhat stable expression trends among the tested time points (64 genes) or a preferential expression at 60 hpi (45 genes). These results are in agreement with the expression profiles that we recently published based on 37 selected HaHV-1 ORFs [38]. Although in-vivo analysis prevents the possibility to have synchronous viral infections due to the variability within cells and animals, this analysis identified viral genes preferentially induced in the early infection and can be used for future comparisons with similar viruses. Up-regulated viral IAPs were reported also for in bivalve species infected with OsHV-1 [25,26,27]. In a detailed study recently published, de Lorgeril et al. (2018) analyzed transcriptomic features of susceptible versus OsHV-1-resistant C. gigas families and showed that the expressions of both endogenous and viral IAPs were significantly up-regulated in susceptible families starting from 24 hpi, while IAPs are not modulated in the resistant family. They suggested that this difference can explain a delayed and inefficient antiviral response of the susceptible oysters [27].

### 3.4. Analysis of DEGs Related to Immune Activities

**Figure 2:**
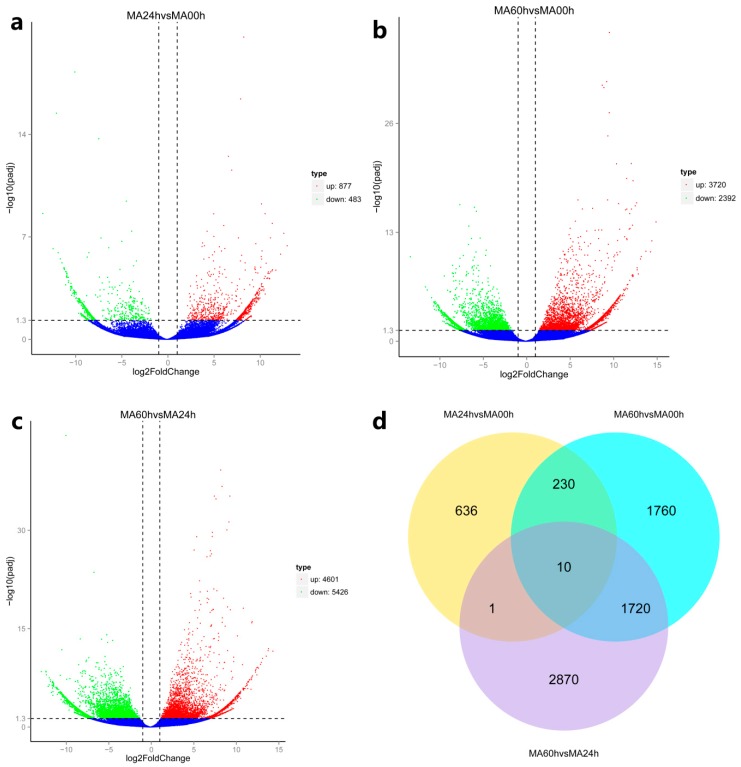
Volcano and Venn plots of differentially expressed genes (DEGs). (**a**) Volcano plots of DEGs comparing MA24h and MA00h groups; (**b**) Volcano plots of DEGs comparing MA60h and MA00h groups; (**c**) Volcano plots of DEGs comparing MA60h and MA24h groups; (**d**) Venn graph of DEGs among the different comparisons. Red spots in panels (**a**), (**b**), and (**c**) depicted over-DEGs, green spots under-DEGs, whereas blue spots referred to non-differentially expressed genes.

Based on differential expression analysis, pairwise comparisons were carried out among the three groups (MA00h, MA24h, and MA60h). A total of 7227 and 6914 unigenes were classified as significantly up-regulated or down-regulated in at least one comparison pair, whereas only 10 up-regulated and 22 down-regulated DEGs are common to all comparisons (Figure 2, Table S6). In details, the comparison between MA60h/MA00h vs. MA60h/MA24h exhibited the highest number (1730) of up-regulated DEGs, followed by that of MA60h/MA00h vs. MA24h/MA00h (240), and between MA60h/MA24h vs. MA24h/MA00h (11). The number of DEGs for each comparison is visualized in Figure 2d. It is interesting that 4601 up-regulated and 5426 down-regulated DEGs were identified when comparing MA60h and MA24h groups, which are higher numbers than those obtained comparing MA60h and MA00h groups (3720 up-regulated and 2392 down-regulated DEGs).

#### 3.4.1. The Delayed Immune Response of Abalone to HaHV-1 Infection

**Figure 3:**
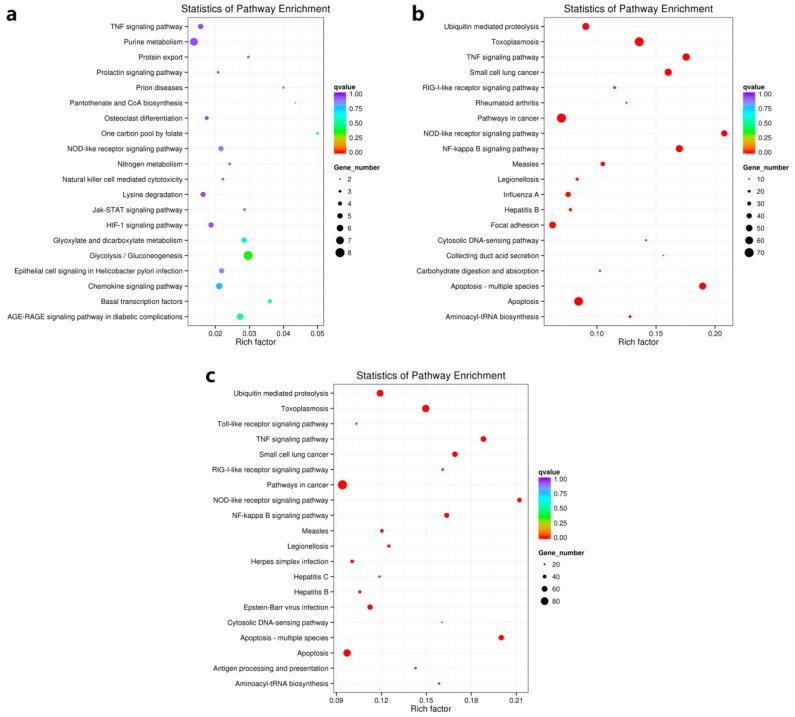
Scatter plot of KEGG enrichment analysis of differentially expressed genes (DEGs) between MA24h and MA00h (**a**), MA60h and MA00h (**b**) and MA60h and MA24h (**c**). Top 20 up-regulated KEGG pathways were displayed. The larger the Rich factor, the greater the degree of enrichment. The value range of *q* value is (0,1). The closer it is to zero, the more significant the enrichment is. Pathways with *q* ≤ 0.05 are defined as pathways that are significantly enriched in DEGs.

Although 877 and 483 unigenes were identified as up-regulated and down-regulated when we compared MA24h vs. MA00h groups, no GO terms with significant changes (*p*-value < 0.05) were identified (Table S7). Accordingly, KEGG pathway enrichment analysis showed no significantly up-regulated pathway (*q*-value < 0.05) (Figure 3a), with only two down-regulated items found significantly enriched (Neuroactive ligand–receptor interaction and ECM–receptor interaction, *q*-value = 0.0395). These results demonstrated that the transcriptome at 24 hpi is similar to the control (0 hpi). No immune-related genes with an increased expression were identified, although the viral DNA increased up to 1.5 × 10^6^ GE/ng^−1^ total DNA and HaHV-1 transcriptome analysis showed the active status of the virus (Table S5). Viral DNA continued to increase, and abalones start to die at 48 hpi, whereas the viral infection burden reaches the peak at 60 hpi. At this point, many up-regulated unigenes associated with antiviral responses were detected. However, this intense but delayed antiviral response was inefficient to protect the animals, and approximately 50% mortality occurred within the following 12 h (from 60 hpi to 72 hpi). This temporal pattern of infection is similar to the one observed for highly susceptible *C. gigas* families, i.e., the deficiency of early antiviral response against OsHV-1 and an intense but inefficient later antiviral responses [27].

The lack of early immune response (before 24 hpi) associated with a considerable viral load is unusual in virally-infected mollusks. This result cannot be explained by the high susceptibility of abalone alone. Actually, the early immune response has been reported even for the *C. gigas* family with the highest susceptibility to OsHV-1 infection, although the level was significantly lower than that of resistant families [16,27]. The lack of immune response observed here may, therefore, be explained by more effective HaHV-1 immune evasion strategies. Higher vertebrate herpesviruses are equipped with sophisticated immune evasion strategies, which include the establishment of latency, molecular mimicry, virus-induced immunosuppression, and modulation of immune recognition structure [9,10]. However, there is little information about the immune evasion strategies deployed by the invertebrate herpesvirus (HaHV-1 and OsHV-1) [16]. Reasonable predictions about these strategies are also impossible due to the low genetic relatedness between them [2,45]. In addition to the immune evasion skills mentioned above, herpesvirus could also hide by adopting more cryptic ways during their spreading. The herpesvirus depends on two fundamental models to enter into the target cells, either by diffusion through the extracellular space (cell-free mode) or by direct cell–cell contact (cell-cell mode) [46,47]. Both mechanisms of viral spread have advantages and disadvantages [47]. The cell-free mode is often inefficient and more vulnerable to the host immune surveillance, because they have to overcome cellular barriers in both donor and target cells. However, the cell-free mode is necessary for spread across long distances and to a new host. The cell-cell mode is very efficient because the barriers could be circumvented by direct cell-to-cell transmission, while the cell-cell mode could be only used within an organism [47]. The cell-cell mode has been recognized as a means to avoid neutralizing antibodies by vertebrate herpesvirus [48]. No data is available about the spreading mechanisms of HaHV-1 within an abalone or among different individuals. However, fewer cases of enveloped HaHV-1 particles were reported [1,5,49,50] compared to that of enveloped OsHV-1 particles [51,52,53]. It is, therefore, possible that the cell-cell spreading was more frequently employed by HaHV-1, and could be partially responsible for the lack of early immune response.

#### 3.4.2. A Rich Set of Immune-Related Pathways in Abalone in Response to HaHV-1 Infection

Between MA60h and MA00h groups, there were 3720 up-regulated and 2392 down-regulated unigenes, respectively. GO term enrichment analysis showed that most of them were classified into two GO categories (biological process and molecular function) with a *p*-value < 0.05 (Table S8). Further KEGG pathway analysis classified the up-regulated DEGs into 261 pathways (Table S9), and 26 of them were found to be significantly up-regulated with a *q*-value < 0.05 (Table 3). The top 20 most up-regulated pathway terms are shown in Figure 3b. Among the 26 significantly enriched pathways, 21 were associated with immunity and disease process and five were related metabolic process. Comparison of MA60h and MA24h groups revealed similar over-expression patterns with MA60h and MA00h groups (Table 3 and Figure 3). These results further indicated that MA24h and MA00h groups have similar transcriptomic profiles and the lack of immunity at 24 hpi.

Ten of the 21 immuno-associated pathways are involved in immunity against virus infection. Seven of these pathways are also well-known in mollusks’ immune defense against pathogenic infections, which include apoptosis, TNF, NOD-like, NF-kappa B, Ubiquitin-mediated proteolysis, RIG-I-like, etc. [16,24,25,26]. Another three pathways, including aminoacyl-tRNA biosynthesis [54], focal adhesion [55], and cytosolic DNA-sensing pathway [56], have been reported to be associated with antiviral response in vertebrate animals. These results indicated that there are a rich set of genes involved in the antiviral response in abalone. The specific role of these pathways in the antiviral process needs further investigation. A considerable number of immune-related genes were also detected in susceptible *C. gigas* compared to resistant individuals after 24 h post-infection [27]. This kind of massive and extensive reprogramming of transcriptomes is unusual for organisms and it will demand a huge amount of energy, which may accelerate the disease process [57]. Accordingly, we identified five up-regulated pathways related to metabolic process, and approximately 50% mortality occurred between 60 and 72 hpi. These results indicate that late activation of immune genes, although in large quantities, was insufficient to conquer the virus and maintain the cellular homeostasis. As a result, several pathways involved in the disease process were over-represented, which indicated that cell and tissue lesions have fully developed, and stood for signals of the immune failure.

A rich gene set associated directly with viral replication and assembly were also identified in challenged abalone at 60 hpi. GO term enrichment analysis identified 11 terms interpreted as viral tegument proteins with a significant *p*-value = 0.0161 compared to the negative control. Additionally, many viral structure proteins associated with viral envelop, membrane, and capsid were also over-expressed at 60 hpi (Table S8). Viral structural proteins are often necessary for the activation of host antiviral response [58]. Therefore, we proposed that during HaHV-1 infection, more and more host cells died, and the accumulated structural proteins of the virus were released out of cells, which should be responsible for the massive enrichment of host immune-related genes.

### 3.5. Validation of RNA-Seq Results by RT-qPCR

Since the detection limit of RT-qPCR is relatively low compared to RNA-Seq, only unigenes with high/middle expression levels could be used for RT-qPCR validation. The relative expression values of RT-qPCR and RNA-seq were described as the log_2_ (fold change). Our results show that 22 out of the total 24 selected genes exhibited a concordant trend between RT-qPCR and RNA-seq expression values. The correlation between the expression levels of RT-qPCR and RNA-seq are shown in Figure S1.

## 4. Conclusions

In the present study, we sequenced the transcriptomes of *H. diversicolor supertexta* infected with HaHV-1 and we investigated virus–host interactions at a molecular level. This work represents the first step towards elucidating the immune mechanisms of abalone against HaHV-1. Unexpected delays of the abalone antiviral response suggested that HaHV-1 has evolved effective strategies to evade early immune surveillance. The over-expressed host genes at the late stages of the infection will provide useful information to understand the molecular mechanisms of the antiviral response of gastropod against dsDNA viruses.

## Figures and Tables

**Table 1:** Summary of the sequencing and assembly results.

Group	Sample	Raw Reads	Clean Reads	Clean Bases	Error (%)	Q20 (%)	Q30 (%)	GC (%)
**MA00h**	MA01	48,448,828	46,980,888	7.05G	0.02	96.66	91.55	44.66
	MA02	46,479,558	44,978,850	6.75G	0.01	97.43	93.28	45.49
	MA03	59,916,192	58,047,078	8.71G	0.02	97.31	93.01	44.71
**MA24h**	MA13	44,682,338	43,431,308	6.51G	0.02	97.2	92.7	43.94
	MA14	49,698,546	48,290,308	7.24G	0.02	97.13	92.52	44.02
	MA15	49,009,274	47,443,952	7.12G	0.02	97.26	92.77	43.23
**MA60h**	MA49	51,712,402	50,051,266	7.51G	0.02	97.17	92.69	46.16
	MA50	41,758,974	40,566,558	6.08G	0.02	96.7	91.62	45.68
	MA51	56,555,122	54,822,210	8.22G	0.01	97.37	93.12	44.81

**Table 2:** Summary statistics of the *Haliotis diversicolor supertexta* transcriptome annotation.

Database	Number of Annotated Unigenes	Percentage of Annotated Unigenes (%)
Annotated in Nr	40,934	19.66
Annotated in NT	71,597	34.39
Annotated in KO	29,322	14.08
Annotated in SwissProt	57,899	27.81
Annotated in Pfam	81,908	39.35
Annotated in GO	82,047	39.41
Annotated in KOG	29,895	14.36
Annotated in all databases	6029	2.89
Annotated in at least one database	131,904	63.37
Total unigenes	208,144	100.00

**Table 3:** Significant up-regulated pathways in *Haliotis diversicolor supertexta* at 60 hpi.

Pathway Name	Pathway ID	MA60h VS MA00h			MA60h VS MA24h		
		Rich Factor	*q* Value	Gene No.	Rich Factor	*q* Value	Gene No.
Apoptosis—multiple species	ko04215	0.19	1.80E-18	54	0.20	7.33E-15	57
TNF signaling pathway	ko04668	0.18	3.21E-18	56	0.19	7.33E-15	60
NOD-like receptor signaling pathway	ko04621	0.21	3.21E-18	48	0.21	4.48E-14	49
NF-kappa B signaling pathway	ko04064	0.17	1.64E-17	55	0.16	2.12E-11	53
Toxoplasmosis	ko05145	0.14	2.02E-17	69	0.15	3.14E-14	76
Small cell lung cancer	ko05222	0.16	1.05E-16	55	0.17	5.89E-13	58
Apoptosis	ko04210	0.08	2.61E-08	67	0.10	9.32E-07	77
Ubiquitin mediated proteolysis	ko04120	0.09	1.12E-07	54	0.12	2.03E-09	71
Measles	ko05162	0.10	3.44E-06	34	0.12	1.74E-05	39
Pathways in cancer	ko05200	0.07	7.87E-06	70	0.09	1.65E-07	94
Aminoacyl-tRNA biosynthesis	ko00970	0.13	4.28E-05	21	0.16	1.45E-05	26
RIG-I-like receptor signaling pathway	ko04622	0.11	0.0003	20	0.16	4.55E-06	28
Influenza A	ko05164	0.08	0.0003	41	0.08	0.0122	44
Cytosolic DNA-sensing pathway	ko04623	0.14	0.0003	15	0.16	0.0007	17
Rheumatoid arthritis	ko05323	0.13	0.0020	14	0.12	0.0469	13
Focal adhesion	ko04510	0.06	0.0024	52	/	/	/
***Collecting duct acid secretion***	***ko04966***	***0.16***	***0.0033***	***10***	/	/	/
Legionellosis	ko05134	0.08	0.0033	24	0.13	1.90E-05	36
***Carbohydrate digestion and absorption***	***ko04973***	***0.10***	***0.0043***	***16***	/	/	/
Hepatitis B	ko05161	0.08	0.0057	25	0.11	0.0007	34
Epstein-Barr virus infection	ko05169	0.06	0.0057	34	0.11	9.32E-07	56
***Cysteine and methionine metabolism***	***ko00270***	***0.09***	***0.0064***	***20***	/	/	/
***Ribosome biogenesis in eukaryotes***	***ko03008***	***0.09***	***0.0098***	***17***	/	/	/
***Starch and sucrose metabolism***	***ko00500***	***0.09***	***0.0115***	***17***	/	/	/
Hepatitis C	ko05160	0.08	0.0157	18	0.12	0.0009	26
Herpes simplex infection	ko05168	0.06	0.049	25	0.10	0.0006	39
Antigen processing and presentation	ko04612	/	/	/	0.14	6.49E-05	26
***Protein processing in endoplasmic reticulum***	***ko04141***	/	/	/	***0.08***	***0.0100***	***48***
Toll-like receptor signaling pathway	ko04620	/	/	/	0.10	0.0082	25
Osteoclast differentiation	ko04380	/	/	/	0.10	0.0162	23
Endocytosis	ko04144	/	/	/	0.08	0.0247	47
Phenylalanine, tyrosine and tryptophan biosynthesis	ko00400	/	/	/	0.20	0.0299	7
***RNA transport***	***ko03013***	/	/	/	***0.08***	***0.0414***	***39***
***Terpenoid backbone biosynthesis***	***ko00900***	/	/	/	***0.15***	***0.0414***	***9***

The items printed in normal were pathways associated with immunity and disease process due to HaHV-1 infection, the pathways printed in bold and italic were pathways associated with metabolic process and proposed to be up-regulated due to the viral infection. The forward slash ‘/’ indicated the absence of data, since thesepathways were not significantly up-regulated at the specific time points compared to the control group (MA00h).

## Supplementary Materials

The following are available online at https://www.mdpi.com/1999-4915/11/4/383/s1, Figure S1. Validation of transcriptomic differentially expressed genes (DEGs) by RT-qPCR. Table S1. Primers used for RT-qPCR validation of RNA-Seq results. Table S2. Functional annotation of the assembled unigenes. Table S3. GO annotation of the *Haliotis diversicolor supertexta* transcriptome. Table S4. KEGG annotation of the *Haliotis diversicolor supertexta* transcriptome. Table S5. Expression of HaHV-1 genes. Table S6. List of differentially expressed genes (DEGs) with annotations and expression values Table S7. GO annotation for up-regulated DEGs between MA24h and MA00h groups Table S8. GO annotation for up-regulated DEGs between MA60h and MA00h groups Table S9. KEGG pathway assignment up-regulated DEGs between MA60h and MA00h groups.
