# Supplementary figures and images for: Dual Transcriptomic Analysis Reveals a Delayed Antiviral Response of Haliotis diversicolor supertexta against Haliotid Herpesvirus-1
Source: Viruses. 2019 Apr 24;11(4):383. doi: 10.3390/v11040383 (PMC6520846; doi:10.3390/v11040383)

Figure S1. Validation of transcriptomic differentially expressed genes (DEGs) by qRT-PCR.

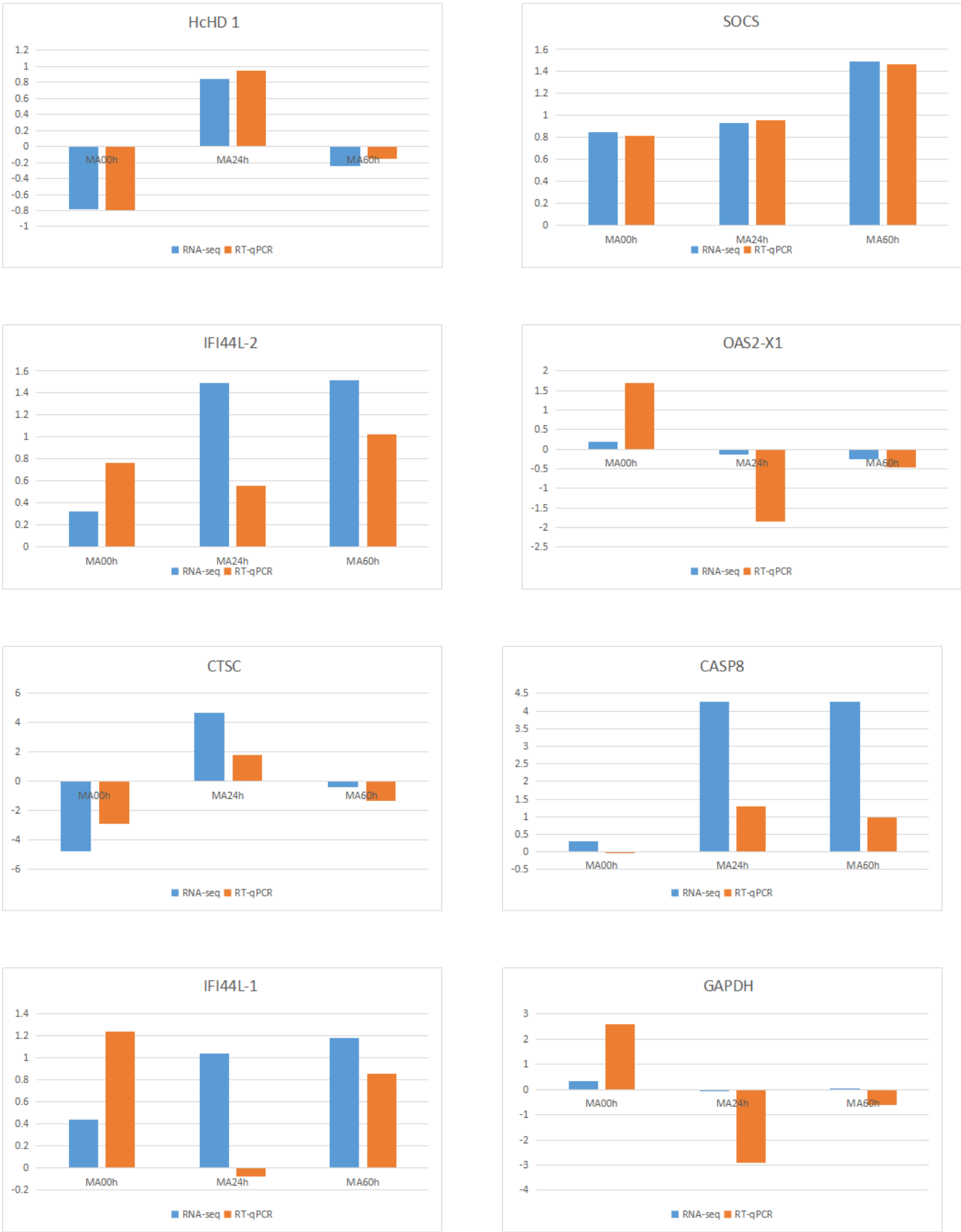

Supplement: Supplementary file 1 [file viruses-11-00383-s001.zip › Figure S1.pdf]
